# Effectiveness, Policy, and User Acceptance of COVID-19 Contact-Tracing Apps in the Post–COVID-19 Pandemic Era: Experience and Comparative Study

**DOI:** 10.2196/40233

**Published:** 2022-10-27

**Authors:** MingXin Liu, SiYu Zhou, Qun Jin, Shoji Nishimura, Atsushi Ogihara

**Affiliations:** 1 Graduate School of Human Sciences Waseda University Tokorozawa Japan; 2 School of Public Health HangZhou Normal University HangZhou China; 3 Faculty of Human Sciences Waseda University Tokorozawa Japan

**Keywords:** COVID-19, contact-tracing app, digital contact tracing, mobile phone

## Abstract

**Background:**

In the post–COVID-19 pandemic era, many countries have launched apps to trace contacts of COVID-19 infections. Each contact-tracing app (CTA) faces a variety of issues owing to different national policies or technologies for tracing contacts.

**Objective:**

In this study, we aimed to investigate all the CTAs used to trace contacts in various countries worldwide, including the technology used by each CTA, the availability of knowledge about the CTA from official websites, the interoperability of CTAs in various countries, and the infection detection rates and policies of the specific country that launched the CTA, and to summarize the current problems of the apps based on the information collected.

**Methods:**

We investigated CTAs launched in all countries through Google, Google Scholar, and PubMed. We experimented with all apps that could be installed and compiled information about apps that could not be installed or used by consulting official websites and previous literature. We compared the information collected by us on CTAs with relevant previous literature to understand and analyze the data.

**Results:**

After screening 166 COVID-19 apps developed in 197 countries worldwide, we selected 98 (59%) apps from 95 (48.2%) countries, of which 63 (66.3%) apps were usable. The methods of contact tracing are divided into 3 main categories: Bluetooth, geolocation, and QR codes. At the technical level, CTAs face 3 major problems. First, the distance and time for Bluetooth- and geolocation-based CTAs to record contact are generally set to 2 meters and 15 minutes; however, this distance should be lengthened, and the time should be shortened for more infectious variants. Second, Bluetooth- or geolocation-based CTAs also face the problem of lack of accuracy. For example, individuals in 2 adjacent vehicles during traffic jams may be at a distance of ≤2 meters to make the CTA trace contact, but the 2 users may actually be separated by car doors, which could prevent transmission and infection. In addition, we investigated infection detection rates in 33 countries, 16 (48.5%) of which had significantly low infection detection rates, wherein CTAs could have lacked effectiveness in reducing virus propagation. Regarding policy, CTAs in most countries can only be used in their own countries and lack interoperability among other countries. In addition, 7 countries have already discontinued CTAs, but we believe that it was too early to discontinue them. Regarding user acceptance, 28.6% (28/98) of CTAs had no official source of information that could reduce user acceptance.

**Conclusions:**

We surveyed all CTAs worldwide, identified their technological policy and acceptance issues, and provided solutions for each of the issues we identified. This study aimed to provide useful guidance and suggestions for updating the existing CTAs and the subsequent development of new CTAs.

## Introduction

### Background

COVID-19 was first identified in Wuhan, Hubei, China, in December 2019 [1,2]. In previous research, the mean reproductive number of COVID-19 was 3.28, which was higher than that of severe acute respiratory syndrome [3]. COVID-19 has a relatively high reproductive number and an initial estimated case fatality rate of 5.6% [4]. As of May 2022, a total of 525 million cases have been confirmed by government agencies worldwide, and more than 6.2 million deaths have been reported [5]. In addition, COVID-19 has considerably affected various service sectors such as tourism [6].

Transmission of COVID-19 is mainly because of airborne transmission of the virus; thus, surface disinfectants, hand hygiene, wearing a mask, ventilation, maintaining social distance, and rapid tracing and notification of potentially infected persons are crucial to prevent infection spread [7,8]. Contact tracing, followed by the prompt isolation of close contacts of an infected individual, is another strategy for preventing virus propagation. Therefore, for tracing infected persons, many countries have launched several digital contact tracing (DCT) methods to supplement manual contact tracing (MCT) [9]. As mobile phones are indispensable in today’s society, several nations have implemented mobile phone–based contact-tracing apps (CTAs) to alert people who have been exposed to the SARS-CoV-2. Currently, despite widespread vaccination in some countries, many parts of the world remain unvaccinated. Even in highly vaccinated countries, COVID-19 continues to cause increased mortality and high levels of infections every 3 to 6 months. The widespread and effective use of CTAs may allow most people to return to their jobs, social events, and family life in capacities similar to those before the pandemic, implying less future morbidity and mortality from COVID-19. In addition, for diseases other than COVID-19 that are transmitted through close contact in the current (eg, monkeypox, Ebola, and COVID-19) and potential future outbreaks, CTA can be used to trace contacts. For example, CTAs have already been used to trace Ebola worldwide, serving as a precedent for mobile phone apps being used to trace contacts for diseases other than COVID-19.

Moreover, because some CTAs register personal information and collect user location data, the protection of personal privacy is an important issue facing CTAs. People worldwide have different attitudes regarding whether governments should protect personal privacy. A study showed that only 6% of people in the United States were very confident, and 25% were somewhat confident that the US government protected data privacy [10]. By contrast, a survey showed that in 2021, a total of 91% of Chinese respondents said that they trusted the Chinese government, the highest rate among the 28 countries surveyed, with a global average of only 52% of respondents saying that they trusted their governments [11]. Another study showed that 37.6% of the Chinese respondents investigated lacked privacy trust in the government [12]. In general, Chinese people have far more trust in their government’s ability to protect their privacy than people in the United States do. This trust may lead to a higher acceptance of CTAs in China than in the United States. Additionally, people in countries with lower economic living standards tend to have lower web-based concerns about private personal information than those living in low-income countries [13]. Differences in people’s privacy concerns and trust in the ability of governments to protect privacy in different countries can lead to large differences in the acceptance of CTAs.

### Literature Review

Previous studies have assessed the effectiveness of CTAs in single countries, including Finland, Switzerland, and Australia [14-16]. Since our study was an international study, it was extremely difficult to assess the effectiveness of all CTAs using the same research method. Therefore, this study focuses on identifying the theoretical and technical issues that may affect the effectiveness of CTAs and offers suggestions for addressing them. A previous study showed that different countries have different data protection policies that prevent most CTAs from being used in other countries like they are used in their own country [17]. However, this study did not mention the extent of interoperability of CTAs or list specific interoperable CTAs. Previous studies in Europe, New Zealand, and Japan have investigated the public acceptance of CTAs and the factors that influence public acceptance using questionnaires [18-20]. Therefore, this study clarifies which CTAs have issues that may affect their public acceptance. Several previous studies have investigated dozens of CTAs. A study selected CTAs from the Google Play Store and Apple App Store, summarized the technology used in the CTAs, and analyzed the advantages and disadvantages of each technology (Bluetooth, GPS, and Wi-Fi) [21]. Another study categorized CTAs by underlying technology and investigated the number of CTA installations and the privacy design and public acceptance [22]. A third study analyzed the different technologies for implementing contact tracing using centralized, decentralized, and hybrid protocols and analyzed privacy issues at the technical level [23]. In addition, several previous studies have compared CTAs around the world [24-26]. However, no study has exhaustively investigated every CTA worldwide or the respective technologies used. CTAs in different countries or continents have various shortcomings, owing to differences in regional characteristics and national policies and the use of different technologies. In contrast to studies that have investigated several CTAs, this study investigated nearly every available CTA and identified common issues across CTAs.

### Study Aims

In this study, we aimed to survey all the CTAs launched in the 197 countries recognized by the United Nations. To the best of our knowledge, this study has investigated the largest number of CTAs and countries to date; we have also categorized and evaluated the features and functions of each CTA by practically using of each CTA, read the description of each CTA, and combined this information with findings from previous research to summarize the known problems and challenges of the CTAs in terms of technical issues, effectiveness, user acceptance, policies, and privacy and given possible solutions for each of these issues. We aimed to provide information for future CTA development and make recommendations for improving CTAs. Governments, health authorities, and technical teams in countries that have already launched CTAs may update and improve the CTAs by following our recommendations, such as extending the tracing distance of Bluetooth-based CTAs and enhancing the cross-border interoperability of CTAs. Countries that have not yet launched CTAs may design them based on the findings of our research.

## Methods

### Overview

The methodology was divided into 2 parts: data collection and data analysis. The data collected in this study included the countries that launched their own CTAs, basic information about these CTAs, and the detection rates of COVID-19 in these countries. For data analysis, we used a literature study to compare our collected data with the results of previous studies; we aimed to determine the problems associated with CTAs and ways to improve them.

### CTA Data Collection Method

To determine the countries that developed COVID-19 apps and collect information on these apps initially, we used Google, Google Scholar, and PubMed to search the keywords of the “country names” of the 197 countries recognized by the United Nations and “COVID-19 contact tracing app,” “digital contact tracing,” “COVID-19 app,” and “mobile applications” in combination. We searched the databases from December 9, 2019 (when COVID-19 was officially confirmed), to March 15, 2022. Only the first 5 pages of the search results were considered because information on the pages thereafter was not relevant to this research. After searching a combination of country names and keywords, we identified the following 3 inclusion criteria based on which the country that was searched for launched its own app. All COVID-19 apps that could be installed during initial data collection were installed and used on the Android system (Samsung Galaxy S20 Ultra) and iOS system (iPhone 11 Pro Max) mobile phones of the first author LMX. The initial collection of COVID-19 apps followed the exclusion criteria for secondary screening. The inclusion and exclusion criteria are shown in Textbox 1.

**Inclusion and exclusion criteria for contact-tracing apps (CTAs); search keywords: “country names,” “COVID-19 contact tracing app,” “digital contact tracing,” “COVID-19 app,” and “mobile apps.”**

**Inclusion criteria**

- Google search results contained information from the government or the health department of the respective country about the COVID-19 app, such as an official website developed by the government.
- Google Scholar and PubMed search results showed published literature indicating that the country launched its own COVID-19 apps.
- Google search results did not contain information from the government or the health department, but there were news reports regarding COVID-19 app use in the country. We searched names of the COVID-19 apps mentioned in news reports on Google, Google Scholar, and PubMed, and the search results satisfied the first 2 criteria.

**Exclusion criteria**

- The app could not be installed with contact-tracing functionality for practical use.
- The app could not be installed or used, but a description of the contact-tracing function of the app could be found on the official website or in literature.
- Apps that were not CTAs, such as the official app launched by Timor-Leste for COVID-19 education.
- Regions that were not among the 197 United Nations–recognized countries were excluded from the study (eg, the British Gibraltar region, despite launching its own CTA, was excluded from this study because the region is not a sovereign state).
- CTAs that were not available in all parts of a country (eg, nonnational CTAs launched by each state government in the United States such as HMushrif, which was only used to trace isolated people and required a bracelet in Oman, and Msafari, which could only trace users in public transportation in Kenya).

Information in languages other than English, Chinese, and Japanese from government and health departments was translated using Google Translate for reading and comprehension. News reports in languages other than English, Chinese, and Japanese were not included in this study during the Google search. Although using each app, it was found that some did not support English, Chinese, or Japanese; for example, many apps from South American countries only supported Spanish. The relevant words or sentences were translated through screenshots using the translation software, DeepL Translate (DeepL SE) and Google Translate.

In this study, information on all CTAs was collected after secondary screening through practical use, consulting official websites, and literature. This information included the technology used by each CTA, whether there was an official website containing information on CTAs, whether they had interoperability with CTAs in other countries, and the COVID-19 detection rate in the country that launched the CTA. The information was first collected through the practical use of the installed CTAs. Regarding CTAs that were installed but could not be used for various reasons (such as the absence of a mobile phone number in the country they were launched) and those that could not be installed, relevant literature and information from the government or health departments was referred to supplement the information gathered by us on these CTAs. The information on CTAs may be outdated in previous literature; thus, even if our results are inconsistent with those of official government or health departments or previous literature, they can be used clinically.

### Data Analysis Through Literature Study

In this research, the information obtained by the method described in the CTA Data Collection Method section was integrated with the findings of previous research and analyzed, and the problems and challenges of the investigated CTAs were summarized.

## Results

### Basic Information of CTAs

**Figure 1:**
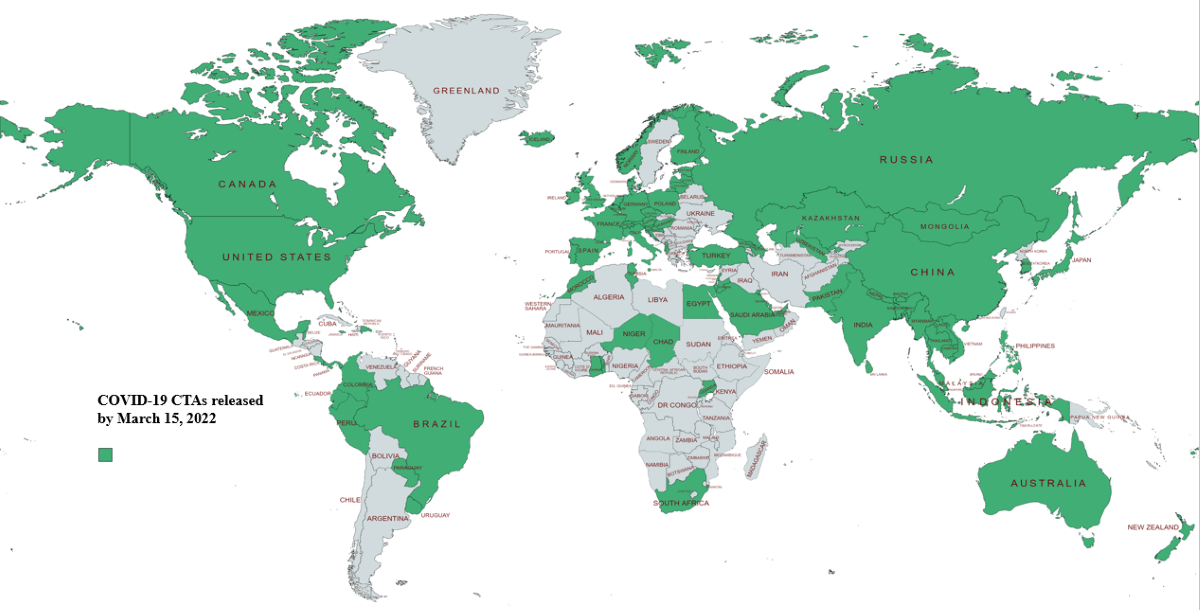
Countries that have released COVID-19 contact-tracing apps (CTAs) as of March 15, 2022 (created with mapchart.net [27]. *Created maps are licensed under a Creative Commons Attribution 4.0 International License [28]).

**Figure 2:**
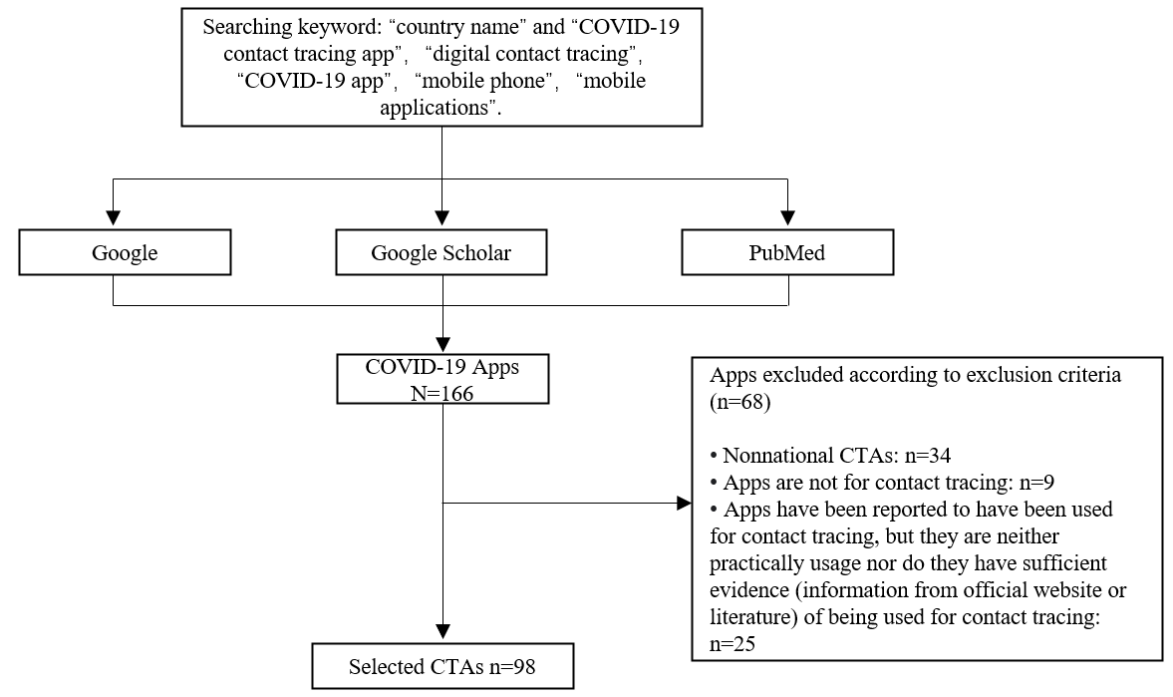
A flow diagram depicting the screening procedure. CTA: contact-tracing app.

After searching keywords through Google, Google Scholar, and PubMed, 64.5 % (127/197) of the countries were found to have launched 166 COVID-19 apps. Excluding 34 nonnational apps, 9 of the remaining apps were not CTAs; 25 apps have been reported to have been used for contact tracing, but they could not be used practically or did not have sufficient evidence (official websites or research papers) regarding contact tracing. Finally, 98 apps developed by 95 countries with sufficient evidence of a contact-tracing function were identified (Multimedia Appendix 1 and Figure 1). China, Saudi Arabia, and Pakistan each had 2 CTAs that were used for contact tracing. A flow diagram depicting the screening procedure is shown in Figure 2.

Among the 95 countries with CTAs, Asian countries had the highest number of CTAs (35/95, 36.8%), whereas Oceania and South America each had the lowest number of CTAs (6/95, 6.3%). Regarding the number of countries with CTAs as a percentage of all countries on a continent, Asia had the highest percentage (35/48, 72.9%), and Africa had the lowest (9/54, 16.7%). The number of CTAs that could be practically used on either Android or iOS systems was 63, accounting for 64.3% (63/98) of all the CTAs. In the remaining CTAs (37/98, 37.8%), the app could not be used because it could not be installed, requiring mobile phone number in a specific country or personal ID, or it could be installed but could not be opened or crashed after opening. After testing all the available CTAs and investigating the introductory information from official websites or previous literature for the nonavailable CTAs, we found that the technologies used for contact tracing could be divided into 3 main categories: Bluetooth, geolocation, and QR code. Bluetooth was the most widely used technology, used in 71 (72.4%) of the 98 CTAs; 35 (35.7%) CTAs used geolocation, and 21 (21.4%) used QR codes (Multimedia Appendix 1). Of the 98 CTAs included in this study, 45 (45.9%) required the registration of personal information, 42 (42.8%) did not, and for 11 (11.2%) CTAs, this information was unknown. The personal information required most often included data such as name, mobile phone number, government identification number, date of birth, and address (Multimedia Appendix 1).

Regarding the interoperability of CTAs among different countries, 17 national CTAs could be operated in different countries. Table 1 shows specific information on the CTAs that had interoperability and the CTAs that could be operated. Of these, 16 were CTAs in EU countries; these CTAs are generally available and can be operated in >10 countries in the European Union. The only CTA other than the CTAs in EU countries that had interoperability was the careFIJI (CTA of Fiji), which can be used in New Zealand.

In addition to collecting information on CTAs, we investigated the estimated COVID-19 infection detection rate (percentage of positives detected to the number of true positives) based on the Institute for Health Metrics and Evaluation model in countries with CTAs; the data were available for 33 countries (Table 2). The highest detection rate was observed in Iceland (52%), and the lowest was observed in Myanmar, Pakistan, Niger, Uganda, and Bangladesh (close to 0%).

Detection rate means percentage of positives detected to the number of true positives. Of the 98 CTAs, 85 (87%) had official websites created by their respective governments, health departments, or developers (Multimedia Appendix 2). In the CTA information available in Multimedia Appendix 2, yes indicates that the official website includes a CTA guide, functions of the CTA, and other CTA-related information, with a total of 70 CTAs; no indicates CTAs with official websites, but without any introduction in official websites, with a total of 15 CTAs; unknown indicates CTAs without official websites, with a total of 13 CTAs. Investigating the download number data for 2022 for each CTA was difficult; only the 2020 download data were available for most CTAs. Of the countries for which download number data were available for 2022, WeChat (a Chinese CTA) had the highest percentage of downloads to the total population at approximately 88.8% (1.28 billion/1.45 billion). The reasons for this high percentage are as follows. (1) WeChat is not only a CTA but also a national chat app in China. (2) In China, it is impossible to enter large public places and move across provinces without the health codes contained in the app. Downloads as a percentage of the total population were, in descending order, Corona-Warn-App (45,820,000/84,324,494, 54.3%; Germany), NHS COVID-19 (31,044,213/68,605,590, 45.3%; United Kingdom), CoronaMelder (5,864,547/17,211,368, 34.1%; the Netherlands), COCOA (37,340,000/125,695,455, 29.7%; Japan), Koronavilkku (1,311,220/5,506,784, 23.8%; Finland), Radar COVID (8,568,514/46,791,314, 18.3%; Spain), and Aarogya Setu (216,800,000/1,407,993,700, 15.4%; India).

**Table 1:** Contact-tracing apps (CTAs) with interoperability and the countries where they can be used.

Country	App name	Countries or regions where apps can be used outside of their own country
Germany	Corona-Warn-App	Belgium, Croatia, Denmark, Estonia, Finland, Germany, Ireland, Italy, Latvia, Lithuania, Malta, the Netherlands, Norway, Poland, Slovenia, Spain, and Switzerland
Switzerland	SwissCovid	Switzerland, Liechtenstein, and Germany
Estonia	HOIA	Belgium, Croatia, Denmark, Estonia, Finland, Germany, Ireland, Italy, Latvia, Lithuania, Malta, the Netherlands, Norway, Poland, Slovenia, Spain, Switzerland, and Cyprus
Spain	Radar COVID	Belgium, Croatia, Denmark, Finland, Germany, Italy, Lithuania, Malta, the Netherlands, Norway, Poland, Slovenia, Ireland, and Latvia
Slovenia	#OstaniZdrav	Austria, Belgium, Croatia, Denmark, Finland, Germany, Ireland, Italy, Latvia, the Netherlands, Poland, and Spain
Croatia	Stop COVID-19	Other European countries
Fiji	careFIJI	New Zealand
Norway	Smittestopp	Denmark, Germany, Ireland, Spain, Latvia, Poland, Cyprus, Croatia, Austria, Finland, the Netherlands, and Belgium
Denmark	Smittestop	Germany, Italy, Ireland, Spain, and Latvia
Finland	Koronavilkku	Other European countries
Ireland	COVID Tracker	Northern Ireland and other European countries
Belgium	Coronalert BE	Germany, the Netherlands, Spain, Ireland, Italy, Denmark, Latvia, Croatia, Poland, and Cyprus
Austria	Stopp Corona	Other European countries
Latvia	Apturi Covid Latvia	Ireland, Italy, Germany, Spain, Denmark, Croatia, Poland, the Netherlands, Belgium, Finland, Austria, Norway, Slovenia, Cyprus, Malta, and Lithuania
Lithuania	Korona Stop LT	Austria, Belgium, Cyprus, Czech Republic, Denmark, Finland, Germany, Ireland, Italy, Latvia, Lithuania, Malta, the Netherlands, Norway, Poland, Slovenia, Spain, and Croatia
Malta	COVID Alert Malta	Other European countries

**Table 2:** Detection rate of countries that launched contact-tracing apps (CTAs).

Country	App names	Detection rate (%)	Date of detection rate data
China	WeChat and Alipay	42	March 14, 2022
Japan	COCOA	17	May 2, 2022
Korea	Corona 100m	26	March 14, 2022
Mongolia	ERSDEL	34	August 16, 2021
Vietnam	PC-Covid	32	May 2, 2022
Thailand	Mor Chana	6	May 2, 2022
Myanmar	SawSaw Shar	0	May 2, 2022
Malaysia	MySejahtera	14	May 2, 2022
Indonesia	PeduliLindungi	1	May 2, 2022
Pakistan	COVID-19 Gov PK and CoCare	0	May 2, 2022
India	Aarogya Setu	1	May 2, 2022
Bangladesh	Corona Tracer BD	0	May 2, 2022
Lebanon	Ma3an	9	May 2, 2022
Iceland	Rakning C-19	52	May 2, 2022
The United Kingdom	NHS COVID-19	35	April 4, 2022
Belgium	Coronalert BE	24	May 2, 2022
Switzerland	SwissCovid	34	May 2, 2022
Hungary	VirusRadar	7	May 2, 2022
Russia	Госуслуги.COVID трекер	6	May 2, 2022
Portugal	StayAway Covid	39	May 2, 2022
Spain	Radar COVID	13	May 2, 2022
Malta	COVID Alert Malta	33	May 2, 2022
Cyprus	CovTracer-EN	25	May 2, 2022
Niger	Niger Contact Tracer	0	May 2, 2022
Uganda	MoH CTC	0	May 2, 2022
South Africa	COVID Alert SA	3	September 27, 2021
New Zealand	NZ COVID Tracer	29	May 2, 2022
Canada	COVID Alert	9	May 2, 2022
The United States	NOVID	13	May 2, 2022
Colombia	CoronApp - Colombia	3	May 2, 2022
Peru	Perú en tus manos	3	May 2, 2022
Brazil	Coronavírus – SUS	5	May 2, 2022
Uruguay	Coronavirus UY	50	April 4, 2022

## Discussion

### Principal Findings

The principle of Bluetooth-based CTAs for contact tracing is that when 2 users with smartphones come into proximity, the CTA records the contact, and the contact record is retained for a certain number of days depending on the presettings of the CTA, usually 14 or 21 days. During this period, if one of the users tests positive for COVID-19, the user will either voluntarily or compulsorily be registered as positive in the CTA. The user who was in close contact with the COVID-19 positive user will be sent an alert notification on their smartphone. The protocols developed based on Bluetooth technology include BlueTrace, DP-3T, Google or Apple Exposure Notification, Pan-European Privacy-Preserving Proximity Tracing, and OpenCovidTrace [9], which differ in the centralization or decentralization in data collection.

**Figure 3:**
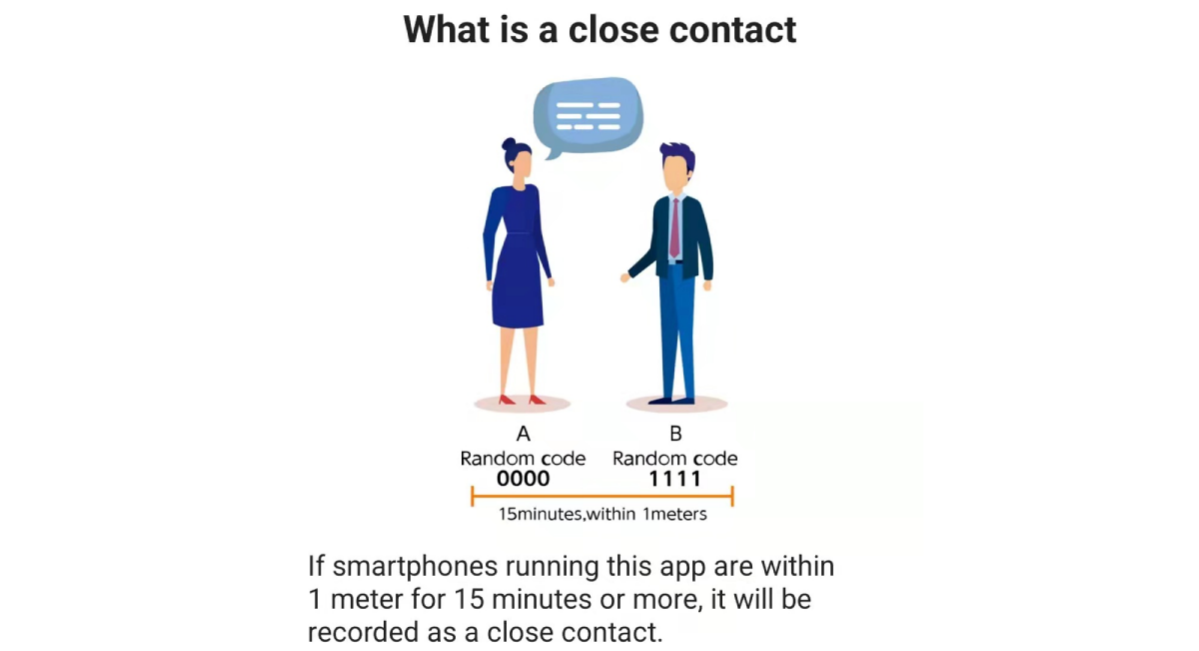
A screenshot within the COCOA app (contact-tracing app of Japan).

Governments can develop their own CTAs based on any of these protocols and determine the specific time and distance for which that proximity will be recorded for the respective CTAs. In this study, 71 CTAs using Bluetooth to record the specific time and distance of contact were investigated; the time and distance data were available for 21 (29.6%) CTAs (Table 3). The shortest contact distance recorded was 1 meter for COCOA (CTA of Japan) and the longest contact distance recorded was 5 meters for Self Safety (CTA of Uzbekistan; Figure 3). The shortest contact time recorded was 1 minute for Self Safety and COVIDSafe (CTA of Australia) and the longest contact time recorded was 20 minutes for VirusRadar (CTA of Hungary). Of the 21 CTAs, 11 (52.4%) set the recorded contact time and distance at 2 meters and 15 minutes. In addition, some countries’ CTAs, such as HaMagen (CTA of Israel), use Wi-Fi–assisted Bluetooth technology for contact tracing.

It should be noted that in Android systems, CTAs using the Google or Apple Exposure Notification protocol request permission to obtain a user’s geolocation but do not use geolocation for contact tracing.

Geolocation-based CTAs trace contact in the following 2 ways. (1) Geolocation records the proximity between mobile phone users and alerts those who have contacted a user who tested positive for COVID-19 and was registered in the CTA, similar to Bluetooth contact tracing. Some CTAs such as Mor Chana (CTA of Thailand) and Aarogya Setu (CTA of India) trace the proximity of contact between 2 users through a combination of GPS and Bluetooth data. This approach can be centralized or decentralized for data collection. (2) WeChat and Alipay (CTAs of China) record the user’s geographic location via a cell tower, and the user’s health code turns red when an infection outbreak occurs in a city or region that the user visited in the past 14 days. This approach is typically centralized for data collection.

**Figure 4:**
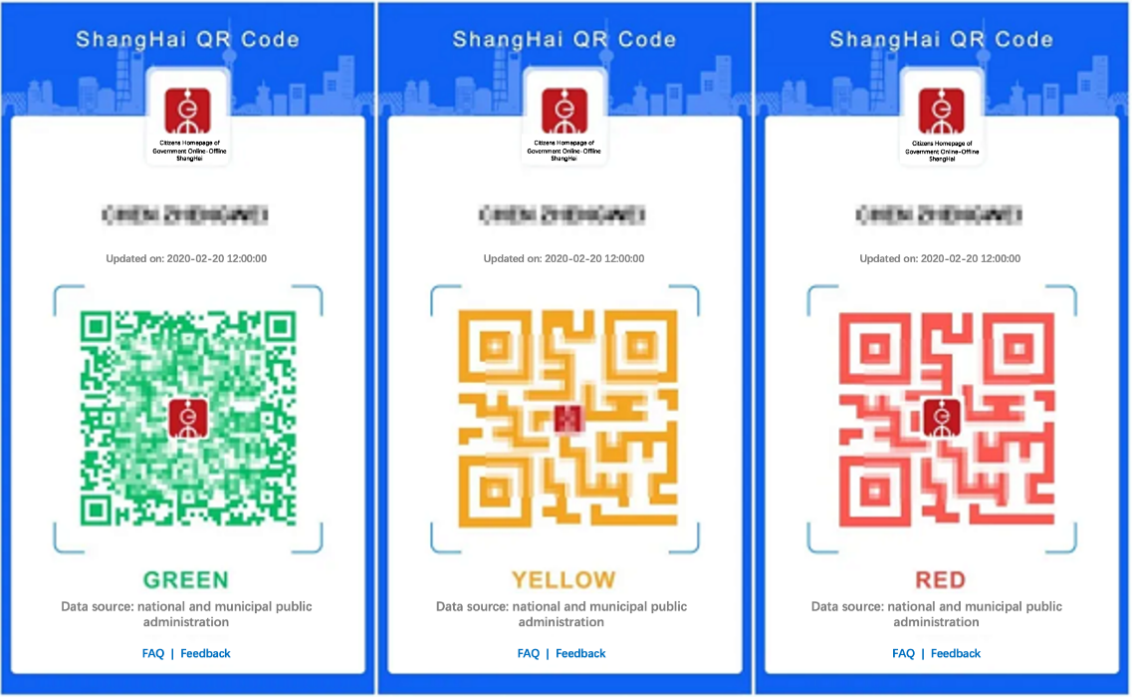
A screenshot of health code used in China.

The principle of CTAs using QR codes to trace contacts is as follows: when a user wants to enter a public place (eg, supermarket, restaurant, or movie theater), the user will either volunteer or be instructed to scan the QR code set up in the public place to record check in and if the user tests positive for COVID-19, the public place that they visited will be classified as a high-risk area. Other users who visited the same area will be notified through an alarm or a red health code in their CTAs (Figure 4).

**Table 3:** Contact-tracing distance and time of contact-tracing apps (CTAs).

Country	App	Contact-tracing distance (m）	Contact-tracing time (min）
Japan	COCOA	1	15
Kazakhstan	Saqbol	2	15
Uzbekistan	Self Safety	5	1
Denmark	Smittestop	1	15
Ireland	COVID Tracker	2	15
France	TousAntiCovid	2	5
The Netherlands	CoronaMelder	1.5	15
Belgium	Coronalert BE	1.5	5
Switzerland	SwissCovid	1.5	15
The Czech Republic	eRouška	2	7
Hungary	VirusRadar	2	20
Latvia	Apturi Covid Latvia	2	15
Lithuania	Korona Stop LT	2	15
Portugal	StayAway Covid	2	15
Spain	Radar COVID	2	15
Tunisia	E7mi	3	N/A^a^
South Africa	COVID Alert SA	2	15
Australia	COVIDSafe	1.5	1
Canada	COVID Alert	2	15
The United States	NOVID	2.7	15
Georgia	Stop Covid	2	15

^a^N/A: not applicable.

### Comparison With Prior Work

Regarding contact-tracing distance and effectiveness, the criteria for the time and distance of proximity set by Bluetooth-based CTAs vary among countries. Regarding the safe social distance for preventing the spread of COVID-19, previous research has shown that 1.6 to 3.0 meters is a safe social distance for the airborne transmission of large droplets exhaled during speech, whereas the distance can reach 8.2 meters if all droplets are considered; furthermore, a social distance of 2 meters is not effective in preventing the spread of infection. [8,29]. Moreover, infectivity varies among different variants of viruses; earlier viruses are usually considered to be the least infectious, while the Alpha variants are considered to be 50% more infectious than the earlier strains, and the Delta variants are considered to be 60% more infectious than the Alpha variants [30]. By contrast, the Omicron variant, recognized by the World Health Organization on November 26, 2021, is considered to be the most infectious variant to date and is >10 times more infectious than earlier strains or 2.8 times more infectious than the Delta variants [31]. Omicron variant transmission was simulated by a Japanese research team in February 2022 using the supercomputer Fugaku. The results showed that while talking without a mask for 15 minutes, the maximum and average rates of infection were approximately 50% and 25%, respectively, for a distance of 2 meters between 2 people, and the rates of maximum and average rates of infection dropped to approximately 10% and 5%, respectively, when the distance was increased to 3 meters [32]. The study also simulated the infection rate of 2 people talking at different times, and the results showed that at a distance of 1 meter, 2 people talking without masks for 15 minutes showed a maximum infection rate of 95% and an average infection rate of 60%; for 6 minutes, they showed a maximum infection rate of 70% and an average infection rate of 30%; for 3 minutes, their maximum and average rates of infection dropped to 30% and 10%, respectively. Therefore, to maintain the infection rate of the Omicron variant at <10%, the tracing distance and time should be set to 1 meter and 3 minutes or 3 meters and 15 minutes. Among the 21 CTAs in Table 3, 14 (66.7%) had a tracing time of 15 minutes and 13 (92.8%) of these had a tracing distance of ≤2 meters. The contact-tracing distance and time settings for these CTAs were 2 meters and 15 minutes, as per the European Center for Disease Prevention and Control guidelines [33]. However, with the drastic reduction in face mask use in some countries, more infectious variants may be transmitted to others at a tracing distance and time much greater than 2 meters and much lesser than 15 minutes, respectively. Thus, current Bluetooth-based CTAs may not be able to trace many potentially infected individuals, because it may have tracing ranges that are too short to identify possible contact and spread. Importantly, in most countries, users who receive a CTA exposure notification are not mandated to quarantine or to undergo polymerase chain reaction (PCR) tests. Instead, with longer tracing distances and shorter tracing times, users can prepare for possible infections after receiving the exposure notification; for example, preparing food and sanitary products before the infection leads to fever and weakness. Thus, setting longer tracing distances and shorter tracing times for CTAs should be preferred.

**Figure 5:**
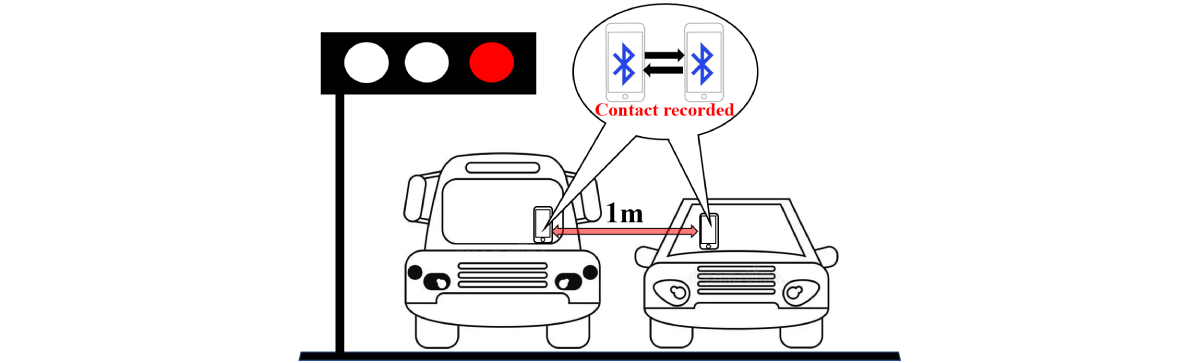
False tracing may happen in 2 adjacent vehicles during traffic jams.

Bluetooth- and geolocation-based contact tracing face many real-life problems that lead to decreased accuracy or false positives. For instance, in a public toilet, although 2 users with CTAs may be at a distance of ≤2 meters, making the CTAs trace contact, the 2 users may actually be separated by a thin wall or two, which could prevent infection [34]. Individuals in 2 adjacent vehicles may face a similar situation during traffic jams (Figure 5). In addition, signal absorption by the human body (when the mobile phone is in the pocket); interference of Wi-Fi signals; and absorption, interference, and diffraction caused by obstacles made of different materials in the signal propagation path can decrease the accuracy of Bluetooth tracing [35-37]. Moreover, different CTA detection rules in railways may lead to a 50% false-alarm rate [37]. Some countries have developed various countermeasures to cope with situations that may lead to decreased accuracy in CTAs. For example, the CTA in Singapore records contacts at a distance of ≥10 meters; therefore, there will often be signals being transmitted through walls, ceilings, and floors. In response, TraceTogether (CTA of Singapore) uploads contact-tracing data to the Ministry of Health, which processes and filters them based on their duration and signal strength to identify only close contacts [38]. HaMagen requests access to body movement data during initial use to avoid false alarm situations when individuals are separated by 2 adjacent vehicles during traffic jams. NOVID, developed at Carnegie Mellon University, uses a combination of ultrasound and Bluetooth. It is the only CTA that uses ultrasound to measure the distance for contact tracing. The NOVID research team experimentally found that at distances<1.82 meter, 103 out of 187 total experimental samples were correctly identified with an accuracy rate of 55.1%, whereas at distances >12 feet, 225 experimental samples were correctly identified 224 times, with an accuracy rate of 99.6%. Moreover, in real life, the tracing accuracy for a distance of <6 feet would be significantly >55.1% [39]. In addition, the ultrasonic method used by NOVID is effective in excluding false positives even in the presence of obstacles [39]. Therefore, ultrasonic technology has great potential for improving the accuracy of CTAs in contact tracing, particularly at long distances and in the presence of obstacles.

The detection rate of COVID-19 is one of the key factors affecting the effectiveness of CTAs. Low detection rates reduce the effectiveness of CTAs because contact tracing is impossible without a confirmed diagnosis, which is a serious challenge for both DCT and MCT. Previous research has shown that contact tracing barely reduces viral propagation when the detection rate is only 13%, and the effectiveness of contact tracing increases significantly when the detection rate increases to 26% or 37% [40]. In June 2020, the average detection rate worldwide was <10% and varied significantly among countries [41]. Therefore, the use of CTAs may not be effective in reducing viral propagation in most countries. Table 2 shows the data for the 33 countries that have developed CTAs wherein detection rates are available [42]. Even in the latter half of 2021 and 2022, only 11 (33.3%) of the 33 countries showed detection rates of >26%, and 3 (9.1%) countries showed detection rates of >37%. There are 16 (48.5%) countries that have not yet reached a detection rate of 13%; in these countries, CTAs can have an extremely limited effect. Currently, PCR testing is free and routine in some countries such as China and Japan. However, the cost of PCR testing varies greatly depending on factors such as the type of laboratory, country or region, and insurance provider [43]. Previous studies have shown that the cost of PCR testing, cost of commuting to a testing site, and time required for PCR testing can make it difficult for people who are willing to be tested [44,45]. Moreover, the convenience and comfort of the sample extraction method affected the willingness to test [45]. In many countries, PCR testing is expensive and difficult to access. This lack of access may lead to CTAs not being able to effectively trace potential infections and eventual getting discontinued. Therefore, making PCR testing free and routine may increase the willingness of users to undergo the test and improve the effectiveness of CTAs. In China, the following methods were used to increase the testing rates. Nationals who did not have a PCR testing certificate within the last 72 hours were not allowed to enter public places such as shopping malls, movie theaters, and public bathrooms and were not allowed to move across provinces by any means of transportation. To reduce costs and improve efficiency, China used a 10-in-1 or 20-in-1 mixed sample, in which samples from 10 or 20 people were tested together in a single sampling tube [46,47]. If the result was negative, it was assumed that all 10 or 20 participants were negative. If the test result was positive, the staff immediately individually isolated 10 or 20 samples in that mixed tube for a temporary period and recollected separate single-tube samples for testing. Furthermore, oropharyngeal swabs were used for routine testing in China and were less painful and more widely accepted than nasopharyngeal swabs [46,47]. These methods have allowed China to maintain a 42% detection rate based on the Institute for Health Metrics and Evaluation model even with a large population of 1.4 billion.

According to our results, of the 197 sovereign countries around the world, 95 (48.2%) have developed 98 apps for contact tracing. However, different countries have different data protection policies that prevent most CTAs from being used in other countries as they are used in their own countries [17]. The CTA interoperability system, which was launched in October 2020 within the European Union, was the first CTA system to remove national operational barriers. The first countries to join this system were Germany, Italy, and Ireland [48]. We investigated CTAs that can be used in other countries as of May 2022; currently, only 17 CTAs can be used in other countries, 16 (94.1%) of which are in the European Union (Table 1). However, with the increasing liberalization of border measures of each country and international travel, the current interoperability of CTAs among countries other than those from Europe does not meet the status quo. International travelers may come into close contact with patients who tested positive for COVID-19 in other countries while traveling on international flights and while traveling in their destination country. However, because CTAs are not interoperable, travelers may not be able to detect transmission or infection resulting from close contact. Since the novel coronavirus has an incubation period of days to weeks, travelers may mistakenly believe that they are not infected until they have significant symptoms. Thus, tourists may spend time in public places for sightseeing, unaware that they have been infected. In this case, if tourists do not restrict their activities in public places, the virus may be further transmitted to more people. Increasing the interoperability of CTAs and removing national barriers among different countries is an important method to avoid cross-country transmission of COVID-19.

CTA policy is no longer stringent. With the increasing number of vaccinations, high asymptomatic rates, and low mortality presented by the Omicron variant compared with initial strains [49], many countries are easing their COVID-19 protection policies, with a trend of relaxation in rules regarding CTAs. For example, according to official information, the QR code–based “check-in” function was abolished in the United Kingdom from February 24, 2022 [50]. Meanwhile, we found through practical use, the QR code scanning function of the previous version of the NHS COVID-19 app (CTA of the United Kingdom) was removed. Moreover, Austria, Cyprus, the Czech Republic, Poland, Denmark, Finland, and Canada have discontinued their CTAs. This implies that CTAs no longer support contact tracing or notifications. However, we believe that this was a premature discontinuation of CTAs because of the following reasons. First, a study in South Africa investigated the asymptomatic rate of different variant infections in 577 health care patients who received a single dose of the Johnson & Johnson vaccine (2.6% for Beta and Delta and 16% for Omicron variants) [49], and another meta-analysis showed an asymptomatic rate of 40% for those infected with the Omicron virus [51]. According to the data, even with vaccination, more than half of the patients infected with Omicron developed symptoms. Therefore, as stated earlier in this section, users who receive notifications of exposure through CTAs can be well prepared for the possibility of symptoms. Especially in countries where medical resources are scarce, users can contact their physicians and reserve hospital beds earlier. Second, in the 2 years since the launch of CTAs, many countries have shown large CTA-using populations. Among the CTAs of countries that have discontinued CTAs, eRouška (CTA of the Czech Republic) has 1.7 million users, reaching 15.9% of the total population [52], and discontinuing CTAs may cause widespread uninstallation of the app. However, it is not possible to predict whether a more infectious variant of COVID-19 with a higher mortality rate or other new infectious diseases will emerge in future; the increase in the number of CTA-installed users is a slow process that may miss the best time for contact tracing. The official website of Smittestopp (CTA of Norway) shows that people in Norway no longer need to register COVID-19 positivity on their CTA. However, the Norwegian Institute of Public Health still supports the CTA and suggests that people keep Smittestopp on their mobile phones in case infection rates start to rise again [53]. We believe that even if the COVID-19 outbreak is contained, it is better to advise people to leave CTAs on their phones, as Norway has done, rather than to uninstall them.

Regarding user acceptance, a study based on a large-scale web-based survey in Japan showed an increase in the level of knowledge related to COCOA (CTA in Japan) to be associated with acceptance of the CTA. Therefore, sufficient knowledge about CTAs, such as the technology of the CTAs for contact tracing, privacy policy, and data storage time, can be effective in encouraging their use among people [20]. Official websites containing information on CTAs are one of the main sources of information for understanding CTAs. In this study, 98 official websites of CTAs were investigated, of which 70 (71.4%) had detailed information, and among the 28 CTAs that lacked sufficient information, 15 (53.6%) had official websites without relevant information, and 13 (46.4%) did not have official websites (Multimedia Appendix 2). Creating understandable information sources for CTAs has great potential for increasing their acceptance among users.

**Figure 6:**
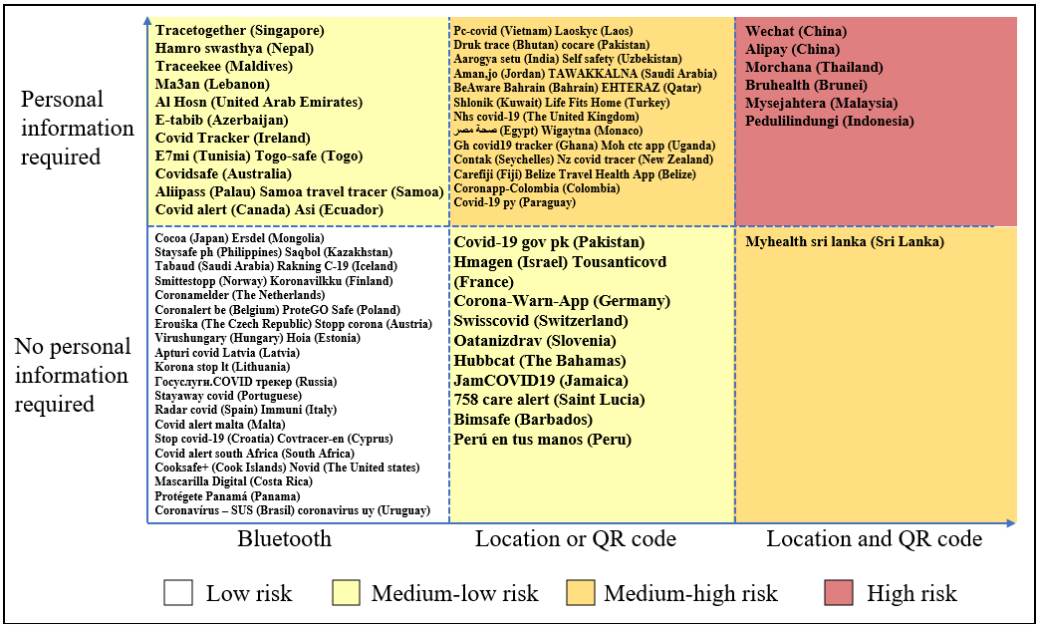
The risk of privacy violation among contact-tracing apps.

Multimedia Appendix 1 lists the 98 CTAs that required personal information to be entered at the time of registration. Compared with other continents, CTAs in Europe generally do not require personal information or require only a small amount of personal information. Of the 28 CTAs in Europe, only the United Kingdom and Ireland require personal information to be entered at the time of registration. It is important to note that the NHS COVID-19 CTA (CTA of the United Kingdom) requires information about the postal code and region of address, and the COVID TRACKER (CTA of Ireland) requires the selection of whether the person is aged >16 years. In contrast to CTAs that require detailed personal information, CTAs in the United Kingdom and Ireland do not require a user’s name, exact address, mobile phone number, or any other detailed information. By contrast, 25 (65.8%) of the 38 CTAs in Asia require personal information; most of which require detailed information such as name and mobile phone number. CTAs that do not require personal information have a lower risk of violating privacy than those that require detailed information. However, as nationals from different countries have different levels of trust in their governments and previous literature shows that people in countries with lower economic living standards are less concerned about the privacy of personal information than people in developed countries [13], nationals of different countries may be differently receptive to CTAs that require personal information. In addition, at the technical level, CTAs based on decentralized Bluetooth technology do not require the user’s location data and thus are most likely to protect the user’s location data. By contrast, location-based CTAs access the user’s location data and thus know where the user is in real time. With QR code–based CTAs, the user’s location is not known when the user does not enter a public place; however, once the user enters a public place, the CTA records “check-ins” at specific locations. Therefore, location-based CTAs and QR code–based CTAs carry the risk of violating users’ location privacy. Figure 6 classifies CTAs by technology and by whether personal information is required, indicating the risk of possible privacy violations. CTAs that use decentralized Bluetooth technology and do not require any personal information have the lowest risk of violating user privacy. Most European CTAs are classified under this category. CTAs that use both location and QR code technologies and require detailed personal information have the highest risk of privacy violation. CTAs in China, Thailand, Brunei, Malaysia, and Indonesia were classified in this category.

### Limitations

We summarized the contact-tracing techniques used in 98 CTAs through practical use and through information on official websites and previous literature. The CTAs we used were the latest versions that could be downloaded; thus, our information on contact-tracing techniques was also up-to-date and reliable. However, after collecting information on the CTAs that could not be used practically through information on official websites and previous literature, we found that some information, especially in the literature, was more than a year old; this information could be inconsistent with the latest version of the CTA. The CTAs marked with letter a in the upper right corner in Multimedia Appendix 1 are those that cannot be used, and the sources of information about these CTAs can be found in Multimedia Appendix 3.

In addition, we used Google Translate and DeepL Translate to understand the content of the literature in languages other than Chinese, English, and Japanese and of CTAs that do not support Chinese, English, or Japanese. Google Translate and DeepL Translate were not always accurate. However, the literature in languages other than Chinese, English, and Japanese and the CTAs in these other languages only account for a very small portion of this study, so they do not have a significant impact on the study. In future studies, we invite researchers who can read other languages to help us.

### Conclusions

For airborne infectious diseases, such as COVID-19, contact tracing is important to reduce viral propagation, and mobile app–based DCT is an effective tool to assist MCT. In this study, we investigated CTAs worldwide; to the best of our knowledge, this is the first study to investigate nearly all CTAs worldwide. We combined the results of this study with those of previous studies to identify common issues in the technology, policy, and user acceptance of CTAs. Most of the current Bluetooth-based CTAs have set the distance and time of tracing contact at 2 meters and 15 minutes; however, considering that several viral variants have stronger infectivity than the initial strains, 2 meters and 15 minutes is not sufficient. Contact-tracing distance and time set at 3 meters and 15 minutes or 1 meter and 3 minutes is preferred. Although some low-income countries with low infection detection rates have launched their own CTAs, the CTAs may not be effective in reducing viral propagation. Currently, only the European Union has developed an interoperable system that allows CTAs to be used in other countries, and outside the European Union, only careFIJI (CTA of Fiji) can be used in New Zealand. Other countries’ CTAs do not have interoperability among different countries. Removing CTAs’ national barriers and increasing interoperability worldwide are critical because countries are gradually opening their borders, and COVID-19 policies are being implemented less strictly. Creating official websites that include information on CTAs would increase their user acceptance and promote installation; 28 (28.6%) of 98 CTAs lacked access to comprehensive and reliable CTA information through official websites. We examined several practical situations that may affect the accuracy of GPS location; 3 CTAs were found to address this lack of accuracy. Among these, the ultrasonic technology used by NOVID was effective in improving the accuracy of the CTA after several rounds of experiments. We found that CTAs have been discontinued in 5 countries since February 2022. We believe that this was a premature discontinuation because COVID-19 has not been eliminated, and it is not possible to predict whether new variants will emerge in the future.

In addition, vaccination has been shown to reduce infection rates, alleviate infection symptoms, and reduce case fatality rates. Therefore, we believe that in countries with widespread vaccination, users do not need extremely stringent measures such as mandatory hospitalization or 14-day self-isolation after receiving notifications of CTA exposure. Overresponse to CTA exposure notifications disrupts both the daily lives of users and the national economy. However, we believe that the greatest benefit of CTAs is that users who receive exposure notifications can prepare for possible infections in advance. For example, users who receive exposure notifications can wear masks to avoid spreading the virus to more people, cancel unnecessary plans to visit public places, make appointments for PCR testing, and prepare medicines in advance to deal with fever and debilitation after infection. Currently, most countries do not mandate that nationals respond upon receiving CTA exposure notification, but the government or health departments should provide guidelines and suggestions on measures to be taken by users who receive an exposure notification. We created a recommendation list for future CTA updates and designs as well as for policy development (Textbox 2).

**Recommendation list for contact-tracing app (CTA) design and policy development.**

**Technical level**

**•** Setting tracing distance and time to 1 meter and 3 minutes or 3 meters and 15 minutes in Bluetooth-based CTAs

• Improving contact-tracing accuracy by using ultrasonic technology, accessing body movement data, and uploading data to the Ministry of Health for processing and filtering

**Policy level**

**•** Removing CTAs’ cross-border barriers and increasing their interoperability worldwide

• Continuing using and maintaining CTAs; encouraging people to use CTAs or at least leave the CTAs installed on their phones

• Providing guidelines and suggestions for users who receive exposure notifications from their CTAs

• Increasing infection detection rate for improving the effectiveness of CTAs by making polymerase chain reaction (PCR) testing free; encouraging people to participate in PCR testing; adopting 10-in-1 mixed samples to reduce costs and improve efficiency; adopting oropharyngeal swabs instead of nasopharyngeal swabs to reduce pain

**Acceptance**

• Creating an official website that includes information on CTAs to increase user acceptance and promote installation

## Appendix

Multimedia Appendix 1 Basic information of all contact-tracing apps.

Multimedia Appendix 2 Official website of all contact-tracing apps.

Multimedia Appendix 3 Information sources of contact-tracing apps that cannot be actually used.

## Abbreviations

CTA: contact-tracing app
DCT: digital contact tracing
MCT: manual contact tracing
PCR: polymerase chain reaction
